# Curcuma reduces kidney and liver damage induced by titanium dioxide nanoparticles in male Wistar rats

**DOI:** 10.22038/AJP.2021.53346.2727

**Published:** 2022

**Authors:** Mandana Shirdare, Fatemeh Jabbari, Maryam Salehzadeh, Nasrin Ziamajidi, Alireza Nourian, Shirin Heidarisasan, Azar Ghavimishamekh, Masoumeh Taheri Azandariani, Roghayeh Abbasalipourkabir

**Affiliations:** 1 *Department of Medical Laboratory, School of Paramedicine, Hamadan University of Medical Sciences, Hamadan, Iran *; 2 *Department of Orthodontics, School of Dentistry, Hamadan University of Medical Sciences, Hamadan, Iran*; 3 *Department of Biochemistry, School of Medicine, Hamadan University of Medical Sciences, Hamadan, Iran*; 4 *Department of Pathology, School of Veterinary Medicine, Bu-Ali Sina University, Hamadan, Iran*; 5 *Neurophysiology Research Center, School of Medicine, Hamadan University of Medical Sciences, Hamadan, Iran*; † * Equal first author*

**Keywords:** Curcuma, Titanium dioxide nanoparticles Toxicity, Wistar rats

## Abstract

**Objective:**

The current study was designed to investigate the protective effects of curcuma caplet against titanium dioxide nanoparticles (nTiO2)-induced damage in liver and kidney of male Wistar rats.

**Materials and Methods:**

Thirty adult (7-8 week old) male rats (200 g) were randomly divided into 5 groups of 6 each. The first and second groups received olive oil and nTiO2 (300 mg/kg body weight) as control and nTiO2 groups, respectively. The third, fourth, and fifth groups received Curcuma at concentrations of 100, 200, and 300 mg/kg body weight in addition to 300 mg/kg body weight of nTiO2, respectively. The treatment was performed through gavage for 3 weeks. Rats' blood was examined for total antioxidant capacity (TAC), total oxidant status (TOS), and malondialdehyde (MDA) levels as well as antioxidant enzymes superoxide dismutase (SOD), and glutathione peroxidase (GPx), and activity of liver enzymes alanine transaminase (ALT), aspartate transaminase (AST), alkaline phosphatase (ALP), lactate dehydrogenase (LDH), and renal factors (urea, uric acid, and creatinine). Histological analyses were also performed to estimate the extent of hepatic and renal injury.

**Results:**

nTiO2-induced liver and kidney damage by decreased serum SOD, GPx, and TAC (p<0.05). Fu

+rthermore, nTiO2 increased serum MDA and TOS, and renal (Creatinine, Urea and Uric acid) and liver parameters (ALT, AST, ALP and LDH) (p<0.05). However, Curcuma treatment was able to moderate these changes dramatically (p<0.05). The results were confirmed by histopathological data.

**Conclusion:**

This study showed the antioxidant properties of curcuma against the side effects of nTiO2.

## Introduction

With the advent of nanotechnology, most nanoparticles can be released into the environment; hence, the effects of nanoparticles on humans and the environment have become a matter of concern for a number of scientists and organizations (Wang et al., 2007 ▶). Meanwhile, the use of these nanoparticles in medicinal products and edible pigments has subjected the general public, especially children to health-related hazards (Weir et al., 2012 ▶). Also, nanoparticles have a long life span in the environment and in the food chain, leading to the persistence of their toxicity (Peter et al., 2004).

 One of these compounds is titanium dioxide nanoparticles. Approximately 95% of titanium is present as titanium dioxide, which is completely insoluble, stable at constant temperatures and non-flammable (Mital and Manoj, 2011). Nowadays, titanium dioxide is employed in the production of paints, cosmetics, targeted drug delivery (Ren et al., 2013 ▶; Du et al., 2015 ▶), photothermal therapy of cancer (Ren et al., 2015 ▶), pigments, optical electronic devices, ceramics, photocatalysts, water and wastewater treatment, and many more applications (Mital and Manoj, 2011; Li et al., 2011 ▶). Due to its small size, it easily crosses the surface of biological membranes and causes an imbalance in the body's antioxidant system, thereby inducing oxidative stress in various organs of the body (Liu and Yang, 2013 ▶). These nanoparticles can cause liver and kidney injury, hepatocyte necrosis, and lung function damage in mice (Wang et al., 2007 ▶). Oxidative stress is caused by an imbalance between the production of free radicals and reactive oxygen species (including superoxide anion, hydroxyl free radical, hydrogen peroxide, etc.) on the one hand and antioxidant defense system on the other. Oxidative stress has detrimental effects on macromolecules, including DNA, proteins, and lipids (Chandra et al., 2015 ▶). From long ago, the significant therapeutic effects of medicinal plants have been considered by researchers (Stener-Victorin, and Lindholm, 2004 ▶). Turmeric is widely used as a food additive. This spice and food coloring is known throughout Asia as a herbal medicine (Bala et al., 2006 ▶). The low cost of this spice and its beneficial effects have made it a necessary part of the daily diet (Aggarwal, 2010 ▶). Studies have shown that turmeric protects the liver against a variety of toxins, including carbon tetrachloride, galactose amine, pentobarbital, acetaminophen, thioacetamide, and aflatoxin (Aggarwal et al., 2003 ▶). Liver protection can be effective in preventing the adverse effects of carbon tetrachloride-induced liver damage in rats (Deshpande et al., 1998 ▶).

This study was designed to investigate the effect of curcuma on titanium dioxide- induced liver and kidney damage. 

## Materials and Methods

**Figure 1:**
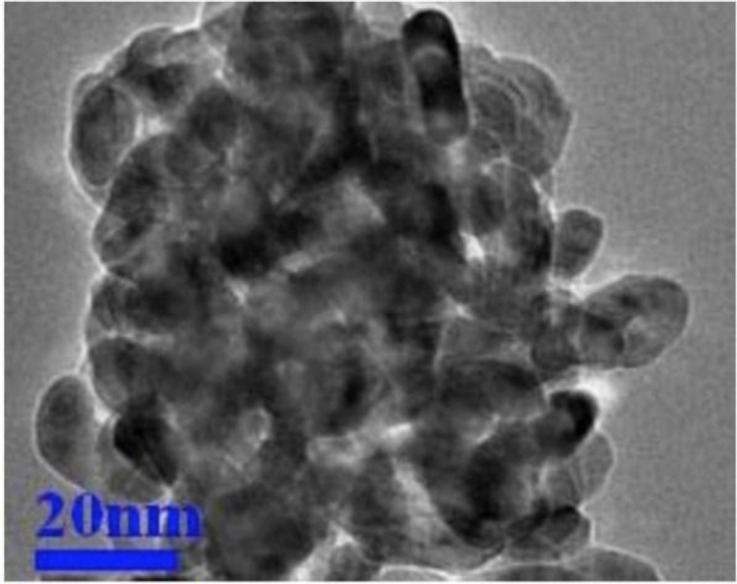
A micrograph of titanium dioxide nanoparticles by transmission electron microscope (TEM)

Titanium dioxide nanoparticles (white, crystal form, size 20 nm, specific surface area 10-45 m^2^/g) were prepared from Iranian Nanomaterials Pioneers Company, NANOSANY (Mashhad, Iran). The characteristics of titanium dioxide nanoparticles (nTiO2) are presented in Table 1 and Figure 1. Curcuma caplet (turmeric caplet) was purchased from Dineh Company (Iran). Each caplet of curcuma contains 450 mg of *Curcuma longa* rhizome powder and 50 mg of *Curcuma longa* extract, which is standardized based on 47.5 mg of curcumin. nTiO2 (Moradi et al., 2019 ▶) and curcuma (Lee et al., 2016 ▶) doses were selected based on previous studies.

**Table 1:** Characteristics of nTiO2 used in the present study

Particle size nm	Specific surface area m^2^/g	Color	Crystal form	pH	Bulk density
20 nm	10-45	White	80 vol% anatase + 20 vol% rutile	5.5-6.0	0.46 g/ml

**Animals and study design**

Thirty adults male Wistar rats aged 7- 8 weeks old (200 g) were randomly divided into 5 groups of 6 each. The control group (C group) received olive oil, the TiO2 group was treated with nTiO2 at 300 mg/kg body weight dissolved in olive oil, the TiO2+ T100 group received nTiO2 at 300 mg/kg body weight + Curcuma caplet at 100 mg/kg body weight dissolved in olive oil, the TiO2+ T200 group received nTiO2 at 300 mg/kg body weight + Curcuma caplet at 200 mg/kg body weight and the TiO2+ T300 group received nTiO2 at 300 mg/kg body weight + Curcuma caplet at 300 mg/kg body weight. The treatment was performed through 1 ml/day gavage for 3 weeks. At the end of the treatment period, the serum of the rats was prepared, and the activity of the liver enzyme including alanine transaminase (ALT), aspartate transaminase (AST), alkaline phosphatase (ALP), and lactate dehydrogenase (LDH) as well as renal factors including urea, uric acid, and creatinine was measured. The animal study was conducted according to the guidelines for the care and use of laboratory animals of the Hamadan University of Medical Sciences, Hamadan, IRAN (IR.UMSHA.REC.1397.523). 

**Biochemistry of serum**

The serum biochemical parameters, creatinine, uric acid, urea, alkaline phosphatase (ALP), alanine transaminase (ALT), aspartate transaminase (AST) and lactate dehydrogenase were measured by an Autoanalyzer (Mindray-BS 480, USA) using Pars Azmun kits (Iran). 

**Serum oxidative stress**

The serum was studied for total antioxidant capacity (TAC), total oxidant status (TOS), and malondialdehyde (MDA) level as well as antioxidant enzymes superoxide dismutase (SOD) and glutathione peroxidase (GPx) according to a previous research (Heidarisasan et al., 2018 ▶).

**Histopathology study**

The liver and kidney of the animals were removed, washed with physiologic serum, fixed in PBS 10%, and finally stained with Hematoxylin and Eosin (H&E). Histological analyses of the tissue sections were performed using a light microscope (ProWay, China) for examining the tissue changes.

**Statistical analysis**

The obtained data were statistically analyzed using SPSS 20 software. The results are presented as mean±standard deviation. One-way analysis of variance (ANOVA) followed by Tukey’s test was carried out to compare the differences of means among the groups. A p<0.05 was appointed as the level of significance. 

## Results

**Effect of nTiO2 and curcuma caplet on body weight of the rats**

Table 2 reports significant increases in weight of the rats during the study (p<0.05). However, there was no meaningful difference between the weight of the animals at the end of the study (p>0.05). 

**Effect of nTiO2 and curcuma caplet on serum biochemical parameters**

According to Tables 3 and 4, treatment with nTiO2 resulted in a significant increase in renal indices creatinine, urea, and uric acid as well as liver function parameters ALT, AST, ALP, and LDH (p<0.05). Treatment with curcuma at 100 mg/kg body weight better than curcuma 300 mg/kg body weight, improved renal parameters (p<0.05). According to the results, curcuma at 300 mg/kg body weight further reduced the serum levels of AST and ALT (p<0.05). However, regarding ALP and LDH, the effect of 100 mg/kg body weight was better than that of 300 mg/kg body weight (p<0.05).

**Table 2:** Effect of titanium dioxide nanoparticles and curcuma caplet on body weight (g) of the rats

Week 4	Week 3	Week 2	Week 1	Groups
282.50±15.82	262.83±11.87	246.33±12.73	211.33±5.75	C
276.50±14.51	260.50±11.04	243.00±10.75	208.16±3.97	TiO2
271.00±28.51	253.66±22.06	236.50±13.33	201.83±6.36	TiO2+T100
272.00±15.92	262.66±18.40	260.83±20.73	211.00±7.32	TiO2+T200
268.83±15.90	249.16±14.45	234.16±8.15	203.16±3.31	TiO2+T300
0.744	0.504	0.021	0.016	p value

All values are expressed as Mean±SD. TiO2 (nTiO2 300 mg/kg bw.); TiO2+T100 (nTiO2 300 mg/kg bw + curcuma caplet 100 mg/kg bw.); TiO2+T200 (nTiO2 300 mg/kg bw + curcuma caplet 200 mg/kg bw.); and TiO2+T300 (nTiO2 300 mg/kg bw + curcuma caplet 300 mg/kg bw.).

**Table 3:** Effect of titanium dioxide nanoparticles and curcuma caplet on kidney parameters in serum of rats

Groups	Creatinine mg/dl	Urea mg/dl	Uric acid mg/dl
C	0.57±0.15	36.33±2.16	1.26±0.023
TiO2	0.94±0.29	75.33±2.73	1.57±0.027
TiO2+T100	0.62±0.19	45.16±3.06	0.70±0.023
TiO2+T200	0.75±0.14	52.16±2.56	0.81±0.021
TiO2+T300	0.83±0.19	60.33±1.75	1.01±0.057
p value	0.000	0.000	0.000

All values are expressed as Mean±SD. TiO2 (nTiO2 300 mg/kg bw.); TiO2+T100 (nTiO2 300 mg/kg bw + curcuma caplet 100 mg/kg bw.); TiO2+T200 (nTiO2 300 mg/kg bw. + curcuma caplet 200 mg/kg bw.); and TiO2+T300 (nTiO2 300 mg/kg bw. + curcuma caplet 300 mg/kg bw.).

**Table 4:** Effect of titanium dioxide nanoparticles and curcuma caplet on serum liver enzymes

Groups	ALP(U/L)	AST(U/L)	ALT(U/L)	LDH(U/L)
C	455.00±4.28	69.66±4.80	47.33±2.06	789.00±3.89
TiO2	639.00±6.54	139.16±3.12	106.17±2.92	1015.33±37.83
TiO2+T100	352.16±3.31	104.83±2.92	83.33±2.58	548.33±3.20
TiO2+T200	379.80±3.86	96.00±1.67	69.40±2.42	595.4±3.93
TiO2+T300	426.33±5.20	81.83±2.13	59.50±1.87	667.50±6.74
p value	0.000	0.000	0.000	0.000

All values are expressed as Mean±SD. TiO2 (nTiO2 300 mg/kg bw.); TiO2+T100 (nTiO2 300 mg/kg bw. + curcuma caplet 100 mg/kg bw.); TiO2+T200 (nTiO2 300 mg/kg bw. + curcuma caplet 200 mg/kg bw.); and TiO2+T300 (nTiO2 300 mg/kg bw. + curcuma caplet 300 mg/kg bw.). ALP: Alkaline phosphatase; AST: Aspartate aminotransferase; ALT: Alanine aminotransferase, and LDH: Lactate dehydrogenase.

**Effect of nTiO2 and curcuma caplet on serum oxidative stress**

As Tables 5 and 6 show, nTiO2 significantly increased TOS and MDA and significantly decreased TAC, SOD, and GPx compared to the control group (p<0.05). Curcumin improved these changes (p<0.05) but did not restore the values to those of the control group. 

**Effect of nTiO2 and curcuma caplet on the tissue structure of liver**

**Figure 2:**
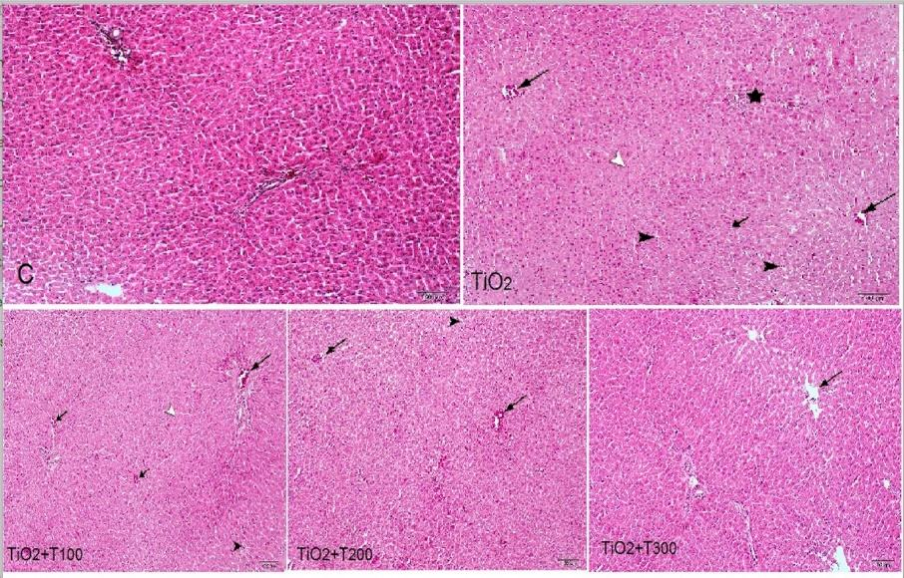
H&E staining photomicrograph of liver tissue of rats (10 x). C: The control group shows tissue structure of the liver containing the normal lobular structure with portal areas, hepatic cords and normal central veins. TiO2 (nTiO2 300 mg/kg bw.) shows portal inflammation with infiltration of white blood cells (stars), central vein hyperemia (large arrows), enlargement of Kupffer cells (small arrow), dilation of sinusoids associated with inflammatory cells accumulation (black arrowhead), and hepatocyte necrosis (white arrow head); TiO2+T100 (nTiO2 300 mg/kg bw. + curcuma caplet 100 mg/kg bw.) shows portal inflammation with venous liver bleeding (larger arrow), lobule center venous bleeding (small arrows), sinusoidal dilatation, and inflammatory cell aggregation (black arrow head), and liver parenchymal cell necrosis (white arrow head), TiO2+T200 (nTiO2 300 mg/kg bw. + curcuma caplet 200 mg/kg bw.) shows center of vein hypertension (arrows) and sinusoidal dilatation (arrowhead), and TiO2+T300 (nTiO2 300 mg/kg bw. + curcuma caplet 300 mg/kg bw.) shows microscopic structure of normal liver lobes, inflammation, hyperemia and enlargement of sinus capillaries

In Figure 2, the microstructural image of the control group shows the tissue structure of the liver consisting of a normal lobular structure with portal areas, hepatic cords, and normal central veins. Treatment with nTiO2 led to liver tissue damage including inflammation, hyperemia, Kupffer cell proliferation, sinusoidal dilatation, and hepatocyte necrosis. Damages were reduced in the groups treated with curcuma at the selected doses. 

**Effect of nTiO2 and curcuma caplet on tissue structure of the kidney**

**Figure 3:**
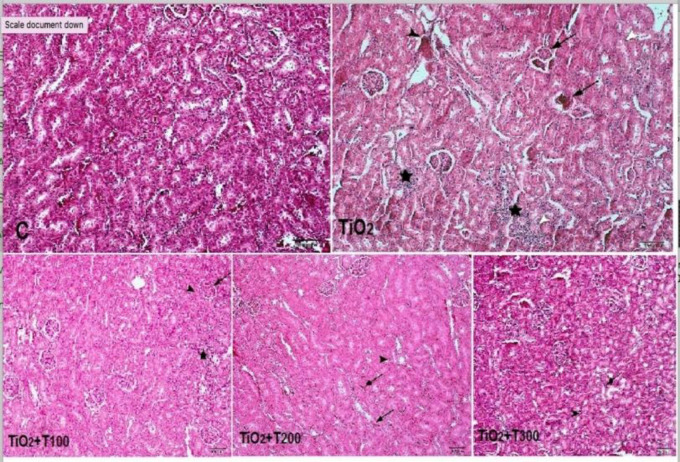
H&E staining photomicrograph of renal tissue of rats (10 x). C: The control group shows the normal tissue structure of the renal glomeruli and nephron tubes. TiO2 (nTiO2 300 mg/kg bw.) shows glomerular wrinkling and atrophy (arrows), vascular hyperemia (black arrowhead), inflammation of the interstitial tissue surrounding the renal tubules with infiltration and accumulation of white blood cells in the area (stars), and necrosis of the epithelial cells of the adjacent renal tubules (white arrows head). TiO2+T100 (nTiO2 300 mg/kg bw. + curcuma caplet 100 mg/kg bw.) shows wrinkles and atrophy of the glomerulus (arrow), inflammation of the interstitial tissue around the tubes with the influence of white blood cells (stars), and necrosis of the epithelial cells of the nearby renal collecting tubules (black arrowhead), TiO2+T200 (nTiO2 300 mg/kg bw. + curcuma caplet 200 mg/kg bw.) shows venous hyperpigmentation (arrows) and mild infiltration of inflammatory cells into the interstitial tissue (arrowhead), TiO2+T300 (nTiO2 300 mg/kg bw. + curcuma caplet 300 mg/kg bw.) shows the microscopic structure of the organ is near-normal and only in some areas of infiltration of white blood cells (arrow) and hyperemia in small blood vessels (arrowhead)

According to Figure 3, the control group presented the normal tissue structure of the kidney, including the renal glomeruli and normal nephron tubes. Glomerular wrinkling and atrophy, vascular hyperemia, interstitial tissue inflammation around the renal tubules, necrosis in the kidney, small blood vessel hyperemia, neuronal apoptosis, and gliosis occurred in the renal tissue of TiO2-treated group compared to the healthy control rats. Treatment with curcumin improved the tissue damage as only a few areas of white blood cell and hyperemia in small blood vessels were observed.

**Table 5:** Effect of titanium dioxide nanoparticles and curcuma caplet on oxidative stress parameters

Groups	TAC (mmole/ml)	TOS (mmole/ml)	MDA (µM/l)
C	8.86±0.25	3.88±0.67	0.213±0.025
TiO2	3.93±0.22	20.68±0.49	0.601±0.022
TiO2+T100	1.67±0.20	2.48±0.19	0.460±0.021
TiO2+T200	2.79±0.36	5.46±0.10	0.386±0.027
TiO2+T300	6.86±0.35	7.64±0.45	0.305±0.028
p value	0.000	0.000	0.000

All values are expressed as Mean±SD. TiO2 (nTiO2 300 mg/kg bw.); TiO2+T100 (nTiO2 300 mg/kg bw. + curcuma caplet 100 mg/kg bw.); TiO2+T200 (nTiO2 300mg/kg bw. + curcuma caplet 200 mg/kg bw.); and TiO2+T300 (nTiO2 300 mg/kg bw. + curcuma caplet 300 mg/kg bw.). TAC: Total antioxidant capacity; MDA: Malondialdehyde; and TOS: Total oxidant status.

**Table 6:** Effect of titanium dioxide nanoparticles and curcuma caplet on serum enzymatic antioxidants

GPx (U/L)	SOD (U/L)	Groups
190.5±5.68	1.93±0.037	C
115.33±4.03	0.66±0.149	TiO2
143.83±3.76	1.27±0.043	TiO2+T100
164.00±4.18	1.51±0.027	TiO2+T200
176.83±3.31	1.71±0.022	TiO2+T300
0.000	0.000	p value

All values are expressed as Mean±SD. TiO2 (nTiO2 300 mg/kg bw.); TiO2+T100 (nTiO2 300 mg/kg bw. + curcuma caplet 100 mg/kg bw.); TiO2+T200 (nTiO2 300 mg/kg bw. + curcuma caplet 200 mg/kg bw.); and TiO2+T300 (nTiO2 300 mg/kg bw. + curcuma caplet 300 mg/kg bw.). SOD: Superoxide dismutase; and GPX: Glutathione peroxidase.

## Discussion

In this study, the toxicity of nTiO2 and protective effects of 100, 200 and 300 of curcuma powder/kg body weight of the rats were evaluated for three weeks in adult Wistar rats based on serum antioxidant enzymes as well as liver and kidney function parameters. We found hepatocyte necrosis in the liver tissue and epithelial cells necrosis in the renal tubules. Previous studies have shown that nTiO2can cause liver damage and induce oxidative stress in rats’ liver (Ma et al,. 2009 ▶). Due to its physicochemical properties, titanium dioxide may cause liver dysfunction. These nanoparticles induce cell death and liver necrosis upon entry into the cell and deployment in the cytoplasm (Sharma et al., 2012 ▶; Rezaei-Zarchi et al., 2012 ▶). nTiO2can enter the body through the mouth, thereby affecting the function of the stomach and intestinal epithelium. In a study, absorption of nTiO2 through the small intestine has been reported (Al-Jubory and Handy, 2013 ▶). Studies have shown that nTiO2 may potentially enter the tissues through the intestine (Brun et al., 2014 ▶). Wang et al. (2007) ▶ reported that two weeks after feeding nTiO2to adult rats, the nanoparticles had a toxic effect at 25 and 80 nm at a dose of 1 g/kg. They performed this experiment through intravenous and intraperitoneal injection of nTiO2and observed nephrotoxicity as renal glomeruli inflammation due to nTiO2accumulation in the kidney. They concluded that nTiO2could penetrate into body tissues and be absorbed by other tissues through the intestines and stomach. It has been shown that in many toxicities, the liver and kidney are more susceptible to damage due to their active role in the metabolism and biochemical changes of environmental pollutants as well as their high blood supply (Mohamed, 2009 ▶). In another study, the effect of nTiO2on rat liver enzyme activity was investigated. Elsewhere, the silver nanoparticles were orally administered to mice and the liver enzymes activity was measured. The results showed elevated serum levels of liver enzymes ALT and AST due to the toxic effect of nanoparticles on the body (Rezaei-Zarchi et al., 2013 ▶). These enzymes are naturally present in the liver cells, and are released into the circulation due to damages to the plasma membrane or cell death, causing elevated serum levels of these enzymes. Thus, a higher serum level of these enzymes is an indicator for assessing the extent of liver cell injury (Liao et al., 2015 ▶). 

Researchers have shown that the Curcuminoids from Curcuma longa L.(Zingiberaceae) is able to protect the skin's epidermal cells from the stress of oxygen radicals (Bonte, 1997 ▶). Based on recent studies, curcumin reduces oxidative damage and apoptosis. Cellular-molecular analysis has shown that exposure of astrocyte and oligodendrocyte from rat glioma to a low dose of curcumin regulated the pentose phosphate pathway by activating glutathione and aldehyde oxidase (Panchal et al., 2008 ▶). Curcumin may lower the levels of serum enzymes such as ALT, AST and LDH which are secreted more during inflammation (Manjunatha and Srinivasan, 2006 ▶). ALT, AST, and ALP are present in the cell cytosol and cell membrane, respectively. These enzymes are released into the blood in response to liver cell destruction. Thus, elevation of these enzymes is a sign of liver cell destruction. ALT and AST are indicators of hepatocyte function while ALP reflects bile duct injury, especially extracellular duct injury (Feldman et al., 2002 ▶; Okechukwu and Segun, 2004 ▶). The results of our study showed that administration of curcuma caused a significant reduction in the acute rise in serum transaminases induced by nTiO2. In a study, Park et al. (2000) ▶ reported the inhibitory effect of turmeric (curcuma) on carbon tetrachloride-induced liver toxicity, which is consistent with the results of the present study. Turmeric has also been shown to improve acute hepatic toxicity due to beta-de-galactose amine and iron, reducing necrosis, and lipid peroxidation in the liver (Park et al., 2000 ▶; Lin SC, 1996 ▶; Ready AC, Lokesh 1999 ▶). In our study, ALP level in the nTiO2 recipient group was significantly elevated, indicating liver damage induced in rats. ALP has been proposed as a tumor marker or marker of liver disease as well as liver injury (Moss DW, 1999 ▶). In our study, the serum level of ALP activity began to decrease from the beginning of the treatment. In addition, LDH levels were significantly lower in the curcuma-treated groups in comparison with the control and titanium dioxide groups, which may be due to the antioxidant and anti-inflammatory properties of curcumin (Sadoughi, 2016 ▶; Sharma, 2007 ▶). Curcumin curbs the activity of inflammatory enzymes, such as cyclooxygenase-2 and lipoxygenase-5, by reducing gene expression of NF-κB as inflammatory parameter (Sharma, 2007 ▶). According to the results, the serum level of urea, uric acid, and creatinine significantly rose in the group treated with titanium dioxide compared to the control group. After treatment with curcuma at 100, 200, and 300 mg/kg, their serum levels were significantly reduced in a dose-dependent manner. The results of this study are consistent with a study where the serum urea, creatinine, and uric acid levels diminished dose-dependently in experimental diabetic groups treated with 100 and 200 mg/kg bw. curcumin (Sadoughi, 2017 ▶). 

It is suggested that different mechanisms are involved in nTiO2 damage such as reactive species oxygen (ROS) production followed by oxidative stress (Li et al., 2014 ▶). In the current study, SOD and Gpx significantly fell in the group treated with titanium dioxide. Lower levels of antioxidant indices can be due to the higher levels of free radicals. SOD has a key role against oxidative damage (Ighodaro and Akinloye, 2017 ▶). The levels of the two enzymes, SOD and GPx, were elevated in groups treated with curcuma at 100, 200, and 300 mg/kg bw. Also, 300 mg/kg bw. of curcuma further increased SOD and GPx and brought them closer to levels observed in the control group. Curcumin suppresses cellular glutathione depletion, increases its levels, and enhances internal antioxidant enzymes activity, while SOD inhibits lipid peroxidation and prevents ROS production (Ognjanović et al., 2010 ▶; Bucak et al., 2010 ▶). Studies by Avci et al. (2012) ▶ and Kalpana et al. (2007) also reported increased levels of CAT, GPx, SOD, and tissue glutathione (GSH) by curcumin in the liver, kidney, and muscles of rats (Avci et al., 2012 ▶; Kalpana et al., 2007). Kalpana et al. (2007) suggested that curcumin could exert its protective effect against nicotine-induced oxidative stress. The results of the present study are in line with the findings of El-Demerdash et al. (2009) ▶ who reported the antioxidant effect of curcumin on oxidative damage induced by sodium arsenite toxicity in rats’ plasma, liver, kidney, lung, testis, and brain through enhanced antioxidant enzymes activity. In a study, in male Wistar rats exposed to curcumin at 100 mg/kg for four weeks, increased sperm count, plasma testosterone level, glutathione and glutathione peroxidase levels, catalase and superoxide dismutase activity, and expression of inflammatory cytokines were observed (Sharma, 2007 ▶). In the present study, curcuma at 100, 200, and 300 mg/kg dose-dependently elevated the level of TAC in comparison to the group treated with nTiO2. Recent studies have shown that curcumin has a phenolic ring and a beta-di-ketone moiety on one molecule, both of which have antioxidant potentials (Palma et al., 2001 ▶; Wang and Huang, 2007 ▶). 

The decreased antioxidant activity along with the increased oxidizing parameters is associated with the toxicity of compounds such as nTiO2. These parameters can be useful in monitoring their toxicity. With the increasing use of nanoparticles in industry, the use of protective and antioxidant herbal compounds such as curcuma can reduce the toxicity caused by nTiO2. The findings of the present study suggest the role of curcuma caplet in controlling oxidative stress indices and preventing lipid peroxidation as well as tissue damage and apoptosis in rats exposed to nTiO2.

## Conflicts of interest

The authors have declared that there is no conflict of interest.
